# Factors Predicting the Success of Adhesiolysis Using a Steerable Catheter in Lumbar Failed Back Surgery Syndrome: A Retrospective Study

**DOI:** 10.3390/jcm10050913

**Published:** 2021-02-26

**Authors:** Ji Yeong Kim, Yong Ho Lee, Subin Yoo, Ji Young Kim, Mina Joo, Hue Jung Park

**Affiliations:** Department of Anesthesiology and Pain Medicine, Seoul St. Mary’s Hospital, College of Medicine, The Catholic University of Korea, Seoul 07345, Korea; apple_queen@naver.com (J.Y.K.); yonghl0915@gmail.com (Y.H.L.); subin.yoo@catholic.ac.kr (S.Y.); chocotruffle@naver.com (J.Y.K.); jma1128@naver.com (M.J.)

**Keywords:** adhesiolysis, failed back surgery syndrome, foraminal stenosis, outer diameter of the catheter, lumbar neuroplasty

## Abstract

Failed back surgery syndrome (FBSS) is a commonly encountered disease after lumbar surgery. There are many cases where it is difficult to choose a treatment because no specific cause can be found. Nevertheless, according to recent reports, adhesiolysis has shown reasonable evidence. However, considering its poor cost-effectiveness, adhesiolysis cannot be used as the first line of treatment. FBSS patients often suffer from chronic pain; accordingly, they become frustrated when this treatment produces a poor response. Therefore, before the procedure, the target group must be selected carefully. We sought to identify the pre-procedure factors predicting the effect of adhesiolysis in FBSS. A total of 150 patients were evaluated and analyzed retrospectively. Of these 150 patients, 69 were classified as responders three months after the procedure (46%). The outer diameter of the catheter during the procedure and grade of foraminal stenosis were correlated with the procedure effect. In conclusion, of the 2.1 mm diameter of the catheter, 1.7 mm of it was used during the procedure, and the milder the foraminal stenosis, the greater the pain reduction effect was three months after the procedure.

## 1. Introduction

A significant proportion of patients undergoing lumbar spine surgery to treat low back pain or radicular pain have experienced symptoms persisting or recurring after surgery [1,2,3,4]. This is called failed back surgery syndrome (FBSS). Although surgical technology has advanced, the surgical success rate remains marginally better after a year than with traditional medication [5]. Surgical failure rates vary according to the literature, but are reported to range from 20% to 40% [1,6,7,8]. They suffer not only from pain, but also from dysfunction, poor quality of life, and mental illness [1,9,10].

Pain physicians often confront FBSS patients in a clinical setting, but treatment is difficult due to complex and heterogeneous pathophysiology [11,12]. Another reason for the difficulty of treating FBSS is the lack of standardized treatment guidelines and the limited use of clinical guidelines by clinicians [13,14,15,16,17]. First, measures to rule out that the cause of pain is not a finding requiring surgical correction such as nerve compression or spine instability should be preceded [18]. Importantly, one of the causes of FBSS to be considered is adhesion, which accounts for 20–36% [19,20,21,22]. Surgery naturally creates adhesions in the epidural space. Once adhesion is made in the epidural space, it interferes with delivery, even if the drug is given by spine injection and causes pain during spine movement [23,24]. Adhesion in the epidural space of the patient can be confirmed by injecting a contrast by caudal injection. The adhesion located at the level corresponding to the symptomatology and image would be likely to be responsible for the symptoms. When the steerable catheter is manipulated in the epidural space and the catheter tip reaches near the adhesion, mechanical adhesiolysis can be performed through side-to-side positioning near the target nerve. After that, when the contrast is injected again, it can be confirmed that adhesion lysis has been successfully performed through the change in the contrast flow. After mechanical adhesiolysis is completed, if the drug is accurately delivered to the vicinity of the target nerve through the catheter tip, it can be expected that the pain will be reduced later. According to the treatment algorithm proposed by Chan et al., it is suggested that in the case of predominant radicular symptoms among FBSS patients, a caudal or root block should be performed first when considering intervention, and if there is no effect, then adhesiolysis could be considered [23,25,26]. Indeed, several systemic reviews have shown that adhesiolysis could be significantly beneficial in FBSS [18,27,28,29]. However, there are no widely accepted guidelines for which adhesiolysis is effective among FBSS patients [14]. We aimed to analyze the preprocedural factors predicting the effect of adhesiolysis, specifically in patients with a history of spine surgery.

## 2. Experimental Section

### 2.1. Subjects

We reviewed the medical records of patients who underwent adhesiolysis using a steerable catheter under the diagnosis of FBSS from 1 January 2013 to 30 June 2020 at the pain clinic of Seoul St. Mary’s hospital. The study protocol was approved by the Ethical Committee of the Seoul St. Mary’s Hospital (KC20RISI0665). This trial was registered on 30 November 2020 at ClinicalTrials.gov (KC20RISI0917). All participants gave informed consent, and this study was conducted in accordance with the Declaration of Helsinki.

All patients received lumbar magnetic resonance image (MRI) prior to undergoing adhesiolysis. When considering adhesiolysis, the pathology that can explain the cause of persistent low leg and/or back pain in lumbar MRI was confirmed in advance. All patients underwent conservative treatment such as exercise and epidural injection before adhesiolysis. The inclusion criteria were as follows: (1) at least 20 years of age; (2) a history of spine surgery; (3) chronic leg pain and/or back pain for at least three months after lumbar surgery; and (4) failure to respond to previous epidural injection combined with exercise and pharmacotherapy. The exclusion criteria were as follows: (1) presence of coagulopathy; (2) presence of malignancy; (3) presence of other causes of symptoms (e.g., peripheral neuropathy); (4) any evidence of central nervous system injury; (5) other procedures such as cervical neuroplasty or nerve block together with lumbar neuroplasty; and (6) follow-up loss before the third month of the procedure. Finally, 150 patient charts were selected and reviewed.

### 2.2. Procedure: Adhesiolysis Using a Steerable Catheter

**Figure 1:**
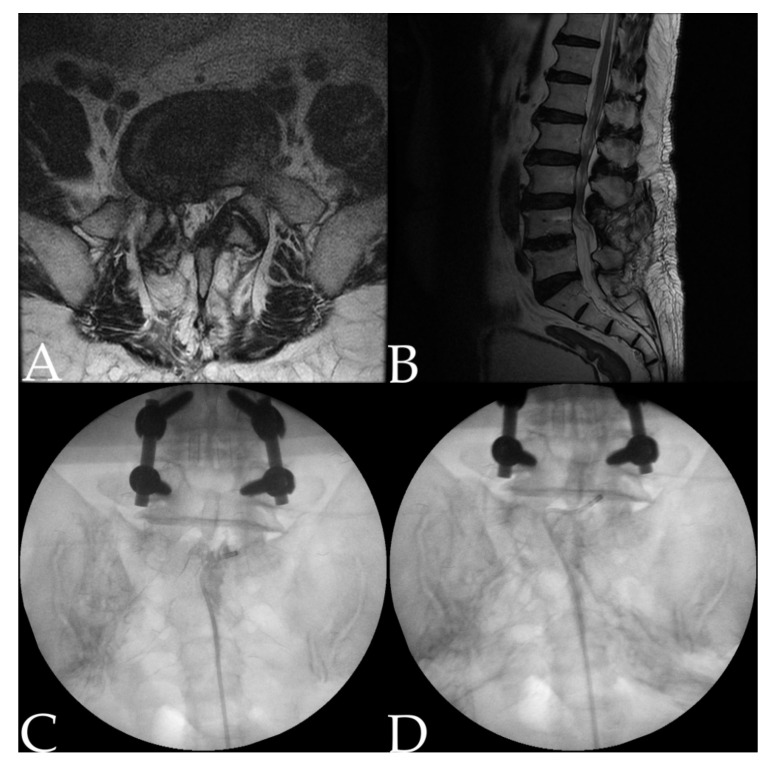
A 59-year-old man underwent L4–5 fusion surgery five years ago for excruciating pain from spinal stenosis. Afterward, the pain improved, and then bilteral leg pain began. He underwent adhesiolysis one year after the onset of symptoms. (**A**) Axial magnetic resonance image (MRI) showed the L5–S1 level with suspected adhesion around the central canal. (**B**) Sagittal MRI showed no specific abnormality at the L5–S1 level. (**C**) Epidurogram confirmed filling defect at the central canal of the L5–S1 level. (**D**) Contrast flow was observed at the L5 vertebra body level after mechanical adhesiolysis with catheter.

Adhesiolysis was performed under fluoroscopic guidance. Before starting the procedure, the patient’s intravenous access should be secured, and pretreatment with antibiotics should be performed. The patient’s vital signs were monitored from the beginning to the end of the procedure. The patient was in a prone position, and a pillow was placed under the abdomen to minimize lumbar lordosis. After the position of the fluoroscope was adjusted so that the lumbosacral area could be viewed in both anteroposterior and lateral views, local anesthetics (1% lidocaine) were infiltrated by determining a needle insertion site around the sacral hiatus. After the guide needle was inserted via the sacral hiatus, approximately 3 mL of diluted contrast medium was injected to obtain the epidurogram of each patient. If contrast flow reveals suspicion of intravascular injection, the needle must be repositioned. The epidurogram was assessed to identify filling defects. Actually, the target level of adhesiolysis is set in advance by taking together the location of the filling defect and the patient’s symptomatic dermatome and lumbar magnetic resonance image (MRI) findings. After determining the goal, the catheter advances through the guide needle (Figure 1).

One of three types of steerable navigation catheters ((1) Episol^®^, GSmedical, Chungcheongbuk-do, South Korea; (2) Biovision^®^, Technologies LLC, Colorado, United States; and (3) STREED plus^®^, Seawon Medi-Tech Co., Ltd., Gyeonggi-do, South Korea) was used, and catheter selection was determined according to the operator’s preference. All catheters used were products that could bend the tip of approximately 1 cm. When the catheter wheel mounted on the operator’s handle was turned, navigation of the catheter tip became easy. The procedure was performed by specialists skilled in neuroplasty. To reach the target site, the catheter was manipulated by pushing, pulling, and rotating. When the catheter tip reached the target, mechanical adhesiolysis was performed through gentle side-to-side positioning of the catheter. At this time, pain may be induced in the same location as the patient felt previously. Then, when 1 cc of diluted contrast was shot, it was confirmed that the filling defect was resolved. The contrast flow appeared in the shape of a nerve root near the neural foramen and an epidural space shape in the central region. When it was confirmed that vascular injection was also excluded, a mixture of 10 cc of 1% lidocaine and 5 mg dexamethasone and 1500 IU hyaluronidase (Hirax^®^, 750 IU/mL, BMIKorea, Gyeonggi-do, South Korea) was divided and injected separately into each target.

Occasionally, in some patients, the catheter was attempted to enter the area where the filling defect was observed on the epidurogram, but the catheter tip could not reach the area, even after repeated attempts. It was clear that if the catheter tip did not reach the target, both mechanical and chemical adhesiolysis could not be performed, so clinical improvement would not be possible. Therefore, only in these cases was a transforaminal epidural injection additionally performed at the level where adhesiolysis was not possible at the end (*n* = 81, 54%).

In the event of suspected complications such as dura mater puncture, the procedure was immediately stopped. Thereafter, the neurological examination was performed multiple times, and only after repeated normal results was the patient discharged from the hospital.

### 2.3. Data Collection

Clinical data such as age, sex, body mass index, duration of symptoms, radiating pain intensity as measured using the numeric rating scale (NRS), and past medical history were obtained through medical records. Whether or not the patient had performed the nerve blocks (e.g., medial branch block, epidural block) during the 3-month follow-up period was also determined through medical records. Simple radiography and MRI were also reviewed to assess the location and severity of lumbar spinal stenosis. The severity was graded based on the standard classification [30,31]. For patients with multilevel central or foraminal stenosis, each level with the greatest stenosis was selected. A total of three types of navigation catheters were used. The Biovision^®^ had an outer diameter of 1.7 mm, the STREED plus^®^ measured 2.1 mm, and the Episol^®^ measured 1.7 mm. In all clinical cases, the outer diameter of the catheter used during the procedure was also identified.

### 2.4. Definition of Treatment Response

The NRS score at three months after adhesiolysis was determined through a chart review. After three months, patients whose NRS decreased by two points compared to before the procedure were classified into the responder group. Patients who received lumbar radiofrequency ablation or a spinal cord stimulator after neuroplasty or transferred to the department of surgery were also defined as nonresponders. Patients with increased oral opioid dose were also classified as nonresponders. According to this definition of response, patients were divided into responders and nonresponders three months after the procedure.

### 2.5. Statistical Analysis

Continuous demographic data were analyzed using the Student’s *t*-test and are presented as the means with standard deviations. Categorical demographic data were analyzed using a chi-square test or Fisher’s exact test and are expressed as numbers and percentages. A two-tailed *p*-value < 0.05 was considered to be statistically significant. All data were analyzed using SPSS version 24.0 (IBM, New York, United States).

## 3. Results

### 3.1. Demographics

**Figure 2:**
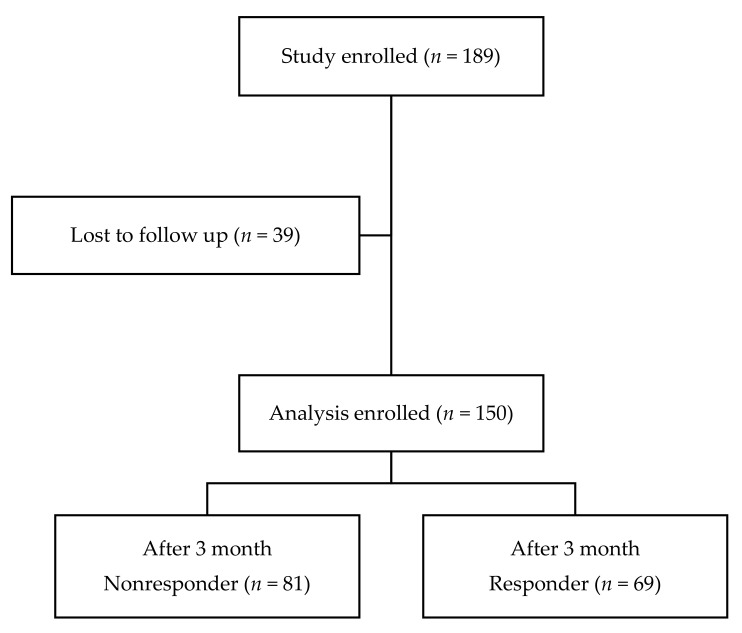
Schematic diagram of the study protocol.

A total of 150 subjects met the inclusion criteria. At the three-month follow-up, 69 subjects were classified into the responder group (46%), and 81 subjects were classified into the nonresponder group (54%) (Figure 2). The basic demographic data of the 150 patients are shown in Table 1. Since the follow up loss rate was significant, baseline analysis was performed between the follow up loss group and the group included in the analysis (Table 2).

### 3.2. Characteristics of the Responders and Nonresponders

The clinical data of nonresponders and responders at three months after the procedure are shown in Table 3. The outer diameter of the catheter (*p* = 0.017) and the foraminal stenosis grade (*p* = 0.039) showed significant results. The catheter diameter used at the time of the procedure was 2.1 mm rather than 1.7 mm, and the milder the degree of foraminal stenosis, the greater the pain three months after the procedure. If the catheter had an external diameter of 2.1 mm, the success rate at three months of the procedure was 56.5%, and for 1.7 mm, the success rate was 37%. In patients with mild cases of foraminal stenosis, the success rate of neuroplasty at three months was 56.4%; in those with moderate cases, it was 40.6%; and in those with severe cases, it was 28.6%. The grade of central stenosis showed no significant relationship with the effect of the procedure (*p* = 0.428). The intervention characteristics were not significantly different between the two groups.

### 3.3. Complications

The complications observed during lumbar neuroplasty are shown in Table 4. Some patients complained of temporary discomfort immediately after the procedure, but all improved within a few days. There were no cases requiring further treatment. There was no correlation between the catheter diameter used during the procedure and the occurrence of dura puncture (*p* = 0.568). When transforaminal epidural injection was performed after adhesiolysis, disc injection rarely occurred. The number of cases with vascular injection was not counted when the needle was readjusted to exclude contrast vascular absorption, so it was very rare. There were no cases of severe neurologic catastrophic complications such as motor weakness.

## 4. Discussion

What is exceptional to note in our report is that the preprocedural effect predictors were presented when adhesiolysis was administered in FBSS, and the analysis was performed by including factors that were not covered in papers that previously presented the effect predictors of adhesiolysis in FBSS [32,33,34].

According to the results of the study, when adhesiolysis was performed in FBSS patients, the milder the foraminal stenosis on lumbar MRI, the better the pain reduction effect after three months of the procedure (56.4%). The foraminal stenosis grade increased, and the outcome of the procedure tended to decrease. However, even with severe foraminal stenosis, 28.6% were successful responders. Therefore, even severe adhesiolysis is difficult to regard as contraindicated. Among the existing studies, no studies have included the grade of central stenosis and foraminal stenosis as analysis factors while analyzing the factors for predicting effects when performing adhesiolysis in the FBSS patient group. Oh et al. [35] reported a preprocedural factor associated with reducing NRS six months after adhesiolysis using a balloon catheter in a patient group without a history of lumbar spine surgery. That study revealed that the foraminal stenosis grade was related to the treatment effect, which is consistent with our report. It is assumed that if foraminal stenosis is severe, it becomes impossible for the navigation catheter to access the pathologic lesion itself where not only adhesiolysis, but also the delivery of medication to the affected area becomes impossible. If the drug is not properly delivered to the affected area, there is no therapeutic effect. On the other hand, although central stenosis is severe, it may be possible to enter the catheter because the space is relatively large compared to the neural foramen. Therefore, the central stenosis grade may be independent of the procedure effect. In central lumbar spinal stenosis, there is a study that reports that the dural sac cross-sectional area and the degree of pain relief after adhesiolysis are not related [36].

In our study, a total of three types of steerable catheters were used. There were two types of 1.7 mm external diameter catheters, Biovision and Episol, and one type of 2.1 mm external diameter catheter, the ST REED Plus. The choice of catheter was determined by the availability and preference of the operator at the time of the procedure. Since the 1.7 mm outer diameter catheter is relatively thin, it has the advantage of easy access even in patients with narrow caudal space. It was estimated that the 2.1 mm outer diameter catheter was relatively rigid so that thick adhesions could be mechanically lysed with stronger power. This is a new report because existing papers have unified the type of catheter as one. However, unlike expectations, there was no correlation between the catheter external diameter and the occurrence of dura puncture.

The basic treatment guidelines of our pain clinic center for FBSS patients are described by Gatzinsky et al. [13], which is based on the multidisciplinary team’s care pathway. First, the patient’s symptoms, physical exam, and radiology were thoroughly analyzed to rule out whether additional surgery was necessary. After ensuring that surgical treatment is not required, pain medication, physiotherapy and rehabilitation should be initiated. If these treatments are not successful, several options for minimally invasive interventional therapies may be tried. When the patient’s symptoms and radiology are synthesized, if the zygapophysial joint is suspected to be the cause of the pain, a medial branch block could be tried, and if a positive response is seen, a radiofrequency rhizotomy can be followed. Alternatively, if spinal stenosis is suspected to be a possible cause of pain from the patient’s symptoms and radiology, epidural block or selective root block may be performed. Epidural adhesiolysis is also included in the reserve option. Reimaging is recommended if new pain occurs at any time or if existing pain acutely worsens during the treatment process. If minimally invasive therapy is not effective, proceed to the stage of invasive spinal cord stimulator or intrathecal morphine pump. Unfortunately, adhesiolysis in FBSS results in pain relief and functional improvement over a relatively short period of six to 24 months [27]. However, compared to implantable technologies, it is easier to perform, has fewer side effects, and has an economic advantage. If a patient with FBSS who visited our pain clinic has undergone a thorough clinical assessment and decided to perform adhesiolysis because of adhesion as the cause, a catheter with an external diameter of 2.1 mm will be used if available. If the foraminal stenosis is mild on lumbar MRI, the patient will be told that the treatment effect will be better. It should be explained to the patient that the duration of the effect may not be long. We will proceed with patient consent after explaining that there may be complications such as subdural injection. It may be necessary to explain in advance that if there is no effect in the future, implantable technologies may be required.

The limitations of this study are as follows: first, follow-up loss was selected as the study exclusion criterion. Follow-up loss may be due to improved symptoms or because the procedure did not work. Therefore, the patient groups with follow-up loss were not included in the analysis. Since the percentage of the follow-up patient group occupied 78 patients, this may be a factor that hinders reaching an accurate conclusion. Second, because the pain duration of the target patient groups was long, patients often did not remember exactly when the pain started. Data collection was based solely on the patient’s statement. The average pain duration value was 72.1 months. As the pain period is long, the accuracy of the pain duration decreases, which can be a factor that prevents accurate conclusions from being reached. In a similar preliminary study that reported that there was a correlation between pain duration and a decrease in pain score six months after the procedure when neuroplasty was performed in FBSS, the patient’s pain duration median value was 36 months [37]. Third, the research design was retrospective and had no control group, so there is a limitation that it can be influenced by other cofounding factors. Fourth, the lack of standardized guidelines for determining that the procedure has been effective can be a problem. The treatment effectiveness criteria are not the same as those of other papers looking at the effect of adhesiolysis, and this may lead to different results.

## 5. Conclusions

Although FBSS is a refractory disease that must be treated with a multidisciplinary approach, adhesiolysis using a steerable catheter could be an effective treatment. When adhesiolysis was performed in the FBSS patient group, the treatment effect was better in cases with mild foraminal stenosis on lumbar MRI. The catheter used during the procedure should be able to reduce the pain more than three months after the procedure when using a 2.1 mm catheter rather than a 1.7 mm external diameter.

## Figures and Tables

**Table 1:** Baseline demographic characteristics.

Parameters	*n* = 150
Age (years)	66.7 ± 12.6
Sex (male/female)	63 (58%)/87 (42%)
Body mass index (kg/m^2^)	24.3 ± 3.6
Duration of pain (months)	72.1 ± 69.6
Pre-procedural NRS	7.0 ± 1.6
Number of previous spine surgeries: 1/2/3 or more	117 (78%)/23 (15.3%)/10 (6.7%)
Concurrent disease: Diabetes/hypertension	38 (25.3%)/71 (47.3%)
Spondylolisthesis	29 (19.3%)
Outer diameter of catheter: 1.7mm/2.1mm	81 (54%)/69 (46%)
Central stenosis: mild/moderate/severe	54 (36%)/24 (16%)/23 (15.3%)
Foraminal stenosis: mild/moderate/severe	55 (36.7%)/32 (21.3%)/14 (9.3%)
Nerve blocks during f/u period after the procedure: Y/N	131 (87.3%)/19 (12.7%)

NRS = Numeric rating scale; f/u = follow up; Y = Yes; N = No; Continuous variables were presented with mean ± standard deviation, and categorical variables were presented with number (percentage).

**Table 2:** Baseline analysis.

	Included Group (*n* = 150)	Follow up Loss Group (*n* = 39)	*p*-Value
Age (years)	66.7 ± 12.5	67.2 ± 13.3	0.827
Sex (male/female)	63 (42%)/87 (58%)	13 (33.3%)/26 (66.7%)	0.325
Body mass index (kg/m^2^)	24.3 ± 3.6	24.9 ± 2.6	0.444
Duration of pain (months)	72.5 ± 69.4	74.2 ± 110.5	0.907
Number of previous spine surgeries: 1/2/3 or more	116 (77.3%)/24 (16%)/10 (6.7%)	27 (69.2%)/7 (17.9%)/5 (12.8%)	0.403
Diabetes	38 (25.3%)	10 (25.6%)	0.969
Hypertension	72 (48%)	13 (33.3%)	0.101
Spondylolisthesis	53 (35.3%)	19 (51.3%)	0.125
Outer diameter of catheter:			0.135
1.7 mm/2.1 mm	80 (53.5%)/70 (46.7%)	26 (66.7%)/13 (33.3%)	
Central stenosis, *n* (%):			0.299
Mild/moderate/severe	43 (43.4%)/28 (28.3%)/28 (28.3%)	5 (25%)/7( 35%)/8 (40%)	
Foraminal stenosis, *n* (%):			0.710
Mild/moderate/severe	48 (49%)/36 (36.7%)/14 (14.3%)	8 (40%)/8 (40%)/4 (20%)	

Continuous variables were presented with mean ± standard deviation, and categorical variables were presented with number(percentage).

**Table 3:** Characteristics of nonresponders and responders at three months after adhesiolysis among patients with lumbar failed back surgery syndrome.

	Nonresponders (*n* = 81)	Successful Responders (*n* = 69)	*p*-Value
Age (years)	66.6 ± 13.1	67.9 ± 11.9	0.265
Sex (male/female)	33 (40.7%)/48 (59.3%)	30 (43.5%)/39 (56.5%)	0.735
Body mass index (kg/m^2^)	24.1 ± 3.4	24.4 ± 3.9	0.750
Pre-procedural NRS	7.1 ± 1.6	7.0 ± 1.6	0.778
Duration of pain (months)	78.5 ± 69.4	64.5 ± 69.6	0.220
Number of previous spine surgeries: 1/2/3 or more	67 (82.7%)/5 (6.2%)/9 (11.1%)	50 (72.5%)/18 (26.1%)/1 (1.4%)	0.951
Diabetes	20 (24.7%)	18 (26.1%)	0.845
Hypertension	37 (45.7%)	34 (49.3%)	0.660
Spondylolisthesis	20 (24.7%)	9 (13%)	0.072
Outer diameter of catheter:			0.017 *
1.7 mm/2.1 mm	51 (63%)/30 (37%)	30 (43.5%)/39 (56.5%)	
Central stenosis, *n* (%):			0.428
Mild/moderate/severe	27 (50.9%)/12 (22.6%)/14 (26.4%)	27 (56.3%)/12 (25%)/9 (18.8%)	
Foraminal stenosis, *n* (%):			0.039 *
Mild/moderate/severe	24 (45.3%)/19 (35.8%)/10 (18.9%)	31 (64.6%)/13 (27.1%)/4 (8.3%)	
Treatment level: 1/2/3 or more	5 (6.2%)/20 (24.7%)/56 (69.1%)	8 (11.6%)/18 (26.1%)/43 (62.3%)	0.249
Treatment location:			0.548
Central only	1 (1.2%)	0 (0%)	
Central with both foramina	77 (95.1%)	65 (94.2%)	
Central with a unilateral foramen (left/right)	3 (3.7%)	4 (5.8%)	
Nerve blocks during f/u period after the procedure: Y/N	72 (88.9%)/9 (11.1%)	59 (85.5%)/10 (14.5%)	0.535

NRS = Numeric rating scale; f/u = follow up; Y = Yes; N = No; Continuous variables were presented with median ± standard deviations and analyzed with a Student t-test. Categorical variables were presented with number(percentage) and analyzed with a Pearson correlation test or Fisher’s exact test. * indicated that *p* value was statistically significant (p < 0.05).

**Table 4:** Complications after adhesiolysis in patients with lumbar failed back surgery syndrome.

Complication	Number
Suspected dura puncture	13
Subdural injection	2
Temporary motor weakness	0
Vascular injection	1
Disc injection	3

## Data Availability

The original data in this study are openly available at https://doi.org/10.6084/m9.figshare.13515935.v2.
